# Ensiling and hydrothermal pretreatment of grass: consequences for enzymatic biomass conversion and total monosaccharide yields

**DOI:** 10.1186/1754-6834-7-95

**Published:** 2014-06-20

**Authors:** Morten Ambye-Jensen, Katja S Johansen, Thomas Didion, Zsófia Kádár, Anne S Meyer

**Affiliations:** 1 Center for BioProcess Engineering, Department of Chemical and Biochemical Engineering, Technical University of Denmark, DTU, DK-2800 Kgs Lyngby, Denmark; 2 Novozymes A/S, Krogshøjvej 36, DK-2880 Bagsværd, Denmark; 3 Danish Plant Breeding Research Division, DLF TRIFOLIUM A/S, Højerupvej 31, DK-4660 Store Heddinge, Denmark

**Keywords:** Silage, Ensiling, Combined pretreatment, Hydrothermal treatment, Grass, Enzymatic hydrolysis

## Abstract

**Background:**

Ensiling may act as a pretreatment of fresh grass biomass and increase the enzymatic conversion of structural carbohydrates to fermentable sugars. However, ensiling does not provide sufficient severity to be a standalone pretreatment method. Here, ensiling of grass is combined with hydrothermal treatment (HTT) with the aim of improving the enzymatic biomass convertibility and decrease the required temperature of the HTT.

**Results:**

Grass silage (*Festulolium* Hykor) was hydrothermally treated at temperatures of 170, 180, and 190°C for 10 minutes. Relative to HTT treated dry grass, ensiling increased the solubilization of dry matter (DM) during HTT and gave increased glucan content, but lower lignin in the insoluble fiber fraction. Ensiling improved glucose yields in the enzymatic hydrolysis of the washed solid fiber fraction at the lower HTT temperatures. At 170°C glucose yield improved from 17 to 24 (w/w)% (45 to 57% cellulose convertibility), and at 180°C glucose yield improved from 22 to 29 (w/w)% (54 to 69% cellulose convertibility). Direct HTT of grass at 190°C gave the same high glucose yield as for grass silage (35 (w/w)% (77% cellulose convertibility)) and improved xylan yields (27% xylan convertibility). The effect of ensiling of grass prior to HTT improved the enzymatic conversion of cellulose for HTT at 170 and 180°C, but the increased glucose release did not make up for the loss of water soluble carbohydrates (WSC) during ensiling. Overall, sugar yields (C6 + C5) were similar for HTT of grass and grass silage at both 170 and 180°C, but at 190°C the overall sugar yield was better for HTT of dry grass.

**Conclusions:**

This study unequivocally establishes that ensiling of grass as a biomass pretreatment method comes with a loss of WSC. The loss of WSC by ensiling is not necessarily compensated for by providing a lower temperature requirement for HTT for high enzymatic monosaccharide release. However, ensiling can be an advantageous storage method prior to grass processing.

## Background

The use of lignocellulosic bioethanol is projected to grow substantially in Europe as a result of EU targets for the transport sector to use 10% renewable energy by 2020 [1] and proposed amendments aimed at promoting non-food derived biofuels [2]. In order to achieve a sustainable biomass supply it is necessary to broaden the range of biomasses used for biofuels and to include the multi-functionality of land use [3].

Temperate grass has excellent potential for bioenergy due to a low energy input, high yield potential, and vast availability in temperate regions of northern Europe. Additionally, growth of perennial grass is multifunctional as grassland areas can benefit the ecological system through the sequestration of carbon into the soil, preventing the agricultural degradation of arable land [4]. Cultivation of temperate grass can therefore make a valuable contribution to sustainable agriculture and land management. Furthermore, carbon sequestration favors the overall carbon balance of the biofuel.

Temperate grasses like tall fescue (*Festuca arundinacea*) and Italian ryegrass (*Lolium multiflorum*) allow the harvest of moist green grass multiple times (three to four cuts) over the season. However, the quantity of DM at harvest constitutes a significant difference between temperate grass and other agricultural residues currently considered as feedstocks for bioethanol, such as wheat straw and corn stover. Wet storage, by means of ensiling, as opposed to field drying (which requires long periods with stable, dry weather conditions or energy demanding indoor drying) is therefore advantageous for temperate grasses. Ensiling is facilitated by low pHs produced by anaerobic solid state fermentation by lactic acid bacteria (LAB), preventing the growth of yeasts, fungi, and other bacteria [5].

Temperate grasses contain considerable amounts of water soluble carbohydrates (WSC), made up of mainly glucose, fructose, sucrose, and fructan [6]. The WSC are important for silage fermentation as they provide the necessary substrate for the LAB, abolishing the need for adding carbohydrates or enzymes for ensiling (this is otherwise required for wheat straw and corn stover) [7,8]. The utilization of the WSC during ensiling may, however, represent a loss of potentially fermentable sugars for processing. Alternatively, the grass would be stored dry, but this requires prolonged field drying where the WSC are prone to losses due to plant respiration, microbial activity, and leaching [5]. The success of field drying is highly dependent on dry weather conditions, as opposed to ensiling.

Pretreatment is considered the most costly step in the conversion of biomass to bioethanol [9-13]. Hence, more efficient pretreatment remains a key challenge in cellulosic bioethanol research. In several studies, ensiling has been shown to improve enzymatic conversion of cellulose compared to dry storage [14-18] and may therefore work as a combined storage and pretreatment method, which can potentially reduce pretreatment costs. However, the enzymatic conversion efficiencies on ensiled biomass have rarely exceeded 50% (converted cellulose/original cellulose) [14,16,18], which is too low to provide enough sugars for a cost efficient ethanol production process [12]. Thus, ensiling cannot compete with the more severe physicochemical pretreatments such as hydrothermal treatment (HTT), steam explosion, dilute acid treatment, or ammonia fiber explosion (AFEX) [19].

HTT uses steam at high temperatures ranging from 170 to 220°C and at corresponding pressure. The pretreatment effect of HTT on biomass is primarily due to autohydrolysis. Water acts as a weak acid, initiating the depolymerization and solubilization of hemicellulose and simultaneous dispositioning of lignin [20,21]. HTT is advantageous to scale up and is applied at a 4 tonne h^−1^ demonstration plant in Kalundborg, Denmark (Inbicon A/S, DONG Energy Denmark) [9].

The combination of ensiling and HTT gives rise to an increased severity of the HTT due to the organic acids in silage, which catalyze biomass hydrolysis [22]. Oleskowicz-Popiel *et al.*[16] previously combined ensiling and HTT using maize (whole crop), rye (whole crop), and a clover grass mixture [16]. They found that HTT increased the ethanol yield of the ensiled biomasses significantly - from 36 to 79% of the theoretical ethanol yield for clover grass. However, ethanol fermentation of hydrothermally treated non-ensiled biomass and any loss of WSC were not investigated, leaving the actual effect of ensiling uncertain. In a recent study on wheat straw we found that ensiling prior to HTT improved the effect of HTT pretreatment at lower temperatures as measured by released glucose and xylose after enzymatic hydrolysis [22]. The glucose and xylose release results showed an improvement of 80 and 81% glucose and xylose yields respectively at 170°C, and 68 and 52% at 180°C.

With the objective of improving the conversion efficiency of ensiled grass fibers, this study examines ensiling of grass in combination with HTT, and assesses whether ensiling may decrease the required HTT operating temperature for obtaining high enzymatic conversion. The main hypothesis tested in this study was that the combination of ensiling and HTT on grass biomass could increase overall sugar yields after enzymatic hydrolysis and that the increase in sugar yields upon enzymatic hydrolysis would exceed the amount of WSC lost to acids during ensiling.

## Results and discussion

### Ensiling

Ensiling of grass proved successful in conserving the biomass by lowering the pH to 4 and limiting the DM loss to 0.6%. Since no considerable changes in the biomass composition were observed after ensiling it can be concluded that no significant degradation of the cellulose and hemicellulose fibers had occurred (Table 1). However, the lack of significant differences in the biomass composition and especially in the hemicellulose levels (calculated as dehydrated xylan and arabinan, Table 1) also implied that no acid hydrolysis of hemicellulosic carbohydrates had occurred during ensiling, in contradiction with the results observed for ensiling in other studies [23,24]. The slightly increased total amount of water extractives after ensiling (which was not statistically significant; Table 1) is most likely a result of some plant cell lysis taking place during ensiling [25].

**Table 1:** Compositions of dry grass and grass silage before HTT

**Biomass**	**Grass**	**Grass silage**
	**(w/w)% of DM**	**(w/w)% of DM**
Glucan	25.2 ± 0.9	24.2 ± 0.3
Xylan	14.1 ± 0.5	14.5 ± 0.2
Arabinan	2.1 ± 0.1	2.4 ± 0.1
Klason lignin	9.3 ± 0.1	9.3 ± 0.1
Ash	8.0 ± 0.5	5.3 ± 0.0
Ethanol Extractives	11.9 ± 0.1	10.5 ± 0.4
H_2_O Extractives	21.1 ± 1.1	25.0 ± 1.5
Residual	8.2	8.9

Numbers are presented as weight percentages of biomass DM, followed by standard deviations (in parenthesis). There were no significant differences row-wise (*p* >0.05) for any of the values for grass and silage. DM, dry matter; HTT, hydrothermal treatment.

The majority (approximately 80% by weight) of the WSC in the dry grass were present as monosaccharides (data not shown) and were mainly glucose and fructose, but xylose, galactose, and arabinose were also detected (Table 2). Only very low amounts of monosaccharides were detected in the silage WSC fraction (Table 2), suggesting that the WSC were utilized as substrates in the anaerobic ensiling fermentation. The compositional data indicate that the WSC were primarily metabolized into lactic and acetic acid (Table 2) during ensiling.

**Table 2:** **Water soluble carbohydrates (WSC) and organic acids in the H**_**2**_**O extracts of dry grass and grass silage**

**Biomass**	**Grass**	**Grass silage**
	**(w/w)% of DM**	**(w/w)% of DM**
Glucose	1.68^a^ ± 0.03	0.32^b^ ± 0.01
Xylose	0.80^a^ ± 0.01	0.00^b^ ± 0.00
Galactose	0.78^a^ ± 0.01	0.00^b^ ± 0.00
Arabinose	0.59^a^ ± 0.00	0.00^b^ ± 0.00
Fructose	1.67^a^ ± 0.12	0.19^b^ ± 0.00
**Total WSC**	**5.52**	**0.51**
Lactic acid	0.10^b^ ± 0.06	6.53^a^ ± 0.30
Formic acid	0.00^a^ ± 0.00	0.00^a^ ± 0.00
Acetic acid	0.19^b^ ± 0.09	1.73^a^ ± 0.08
Propionic acid	0.01^a^ ± 0.01	0.04^a^ ± 0.00
**Total organic acids**	**0.30**	**8.30**

Numbers are presented as weight percentages of biomass DM, followed by standard deviations (^a^ and ^b^). The results in each row are grouped according to significance (*P* <0.05%), where ^a^ is significantly higher than^ b^ (similar superscript letters indicate no statistically significant difference). DM, dry matter; HTT, hydrothermal treatment; WSC, water soluble carbohydrates.

### Hydrothermal treatment

After HTT pretreatment, each biomass sample was separated into a liquid and a solid fraction. The solubilization of DM during HTT pretreatment increased with the HTT temperature and was higher for the grass silage at each HTT temperature (Tables 3 and 4). The glucan content in the solid fraction of each biomass sample also increased with the HTT temperature and the glucan levels in the HTT pretreated grass silage fibers were consistently higher than those in the HTT treated dry grass fibers at all HTT temperatures (Table 3). In contrast, the lignin levels and the total DM recovery were consistently lower in the HTT treated grass silage fiber samples than those in the HTT treated dry grass fibers (Table 3). Xylan levels were similar in the HTT treated grass silage fiber samples and the HTT treated dry grass fibers (Table 3).

**Table 3:** Compositions of solid fractions after HTT treatments at different temperatures (°C)

**Biomass**	**Grass**			**Grass silage**		
**HTT temperature**	**170**	**180**	**190**	**170**	**180**	**190**
	**(w/w)% of DM**			**(w/w)% of DM**		
Glucan	31.2^d^ ± 0.45	33.0^d^ ± 0.16	35.0^cd^ ± 0.43	36.9^c^ ± 0.43	40.6^b^ ± 0.00	42.8^a^ ± 1.10
Xylan	18.7^a^ ± 0.40	17.1^b^ ± 0.05	10.3^c^ ± 0.09	19.1^a^ ± 0.09	17.5^b^ ± 0.35	10.0^c^ ± 0.21
Arabinan	2.7^a^ ± 0.01	1.8^b^ ± 0.07	1.0^d^ ± 0.00	2.7^a^ ± 0.00	1.3^c^ ± 0.01	0.5^e^ ± 0.01
Klason lignin	18.8^a^ ± 0.59	19.0^a^ ± 0.67	17.0^a^ ± 0.58	13.8^b^ ± 0.58	13.5^b^ ± 0.07	13.9^b^ ± 0.30
Ash	7.4^ab^ ± 0.52	8.2^a^ ± 0.18	8.4^a^ ± 0.11	7.8^ab^ ± 0.11	6.8^b^ ± 0.09	6.8^b^ ± 0.02
Ethanol Extractives	11.8^c^ ± 0.35	16.4^b^ ± 0.49	22.8^a^ ± 0.86	15.4^b^ ± 0.16	18.2^b^ ± 0.76	24.5^a^ ± 0.61
Residual	9.5	4.4	5.6	4.2	2.2	1.4
**DM recovery in solid fraction**	**75.3**	**69.6**	**61.6**	**67.5**	**61.5**	**58.8**

Numbers are presented as weight percentages of DM in the HTT solid fraction, followed by standard deviations (except for ‘**DM recovery in solid fraction**’, which is the percentage of DM left in solid fraction after HTT pretreatment, based on mass balance after HTT pretreatment. Different superscript letters indicate significantly different values (*P* <0.05) row-wise; (similar superscript letters indicate no statistically significant difference). DM, dry matter; HTT, hydrothermal treatment.

**Table 4:** Sugars and organic acids in the HTT liquid fraction

**Biomass**	**Grass**			**Grass silage**		
**HTT temperature**	**170**	**180**	**190**	**170**	**180**	**190**
	**(w/w)% of DM**			**(w/w)% of DM**		
Glucose	9.7^a^	10.5^a^	10.1^a^	1.8^c^	2.4^b^	2.3^b^
Xylose	3.8^f^	8.4^d^	17.2^a^	5.8^e^	12.4^c^	14.9^b^
Galactose	1.4^d^	2.1^b^	2.6^a^	1.4^d^	2.0^b^	1.7^c^
Arabinose	2.1^d^	3.1^b^	3.8^a^	2.5^c^	2.9^b^	2.4^c^
Fructose	3.0^a^	2.9^a^	2.0^b^	0.5^c^	0.6^c^	0.3^d^
**Total sugars**	**20.0**	**27.1**	**35.7**	**12.1**	**20.3**	**21.7**
Lactic acid	0.10^b^	0.39^b^	0.22^b^	11.51^a^	11.97^a^	11.27^a^
Formic acid	0.83^d^	0.98^c^	1.25^b^	2.31^a^	2.35^a^	2.51^a^
Acetic acid	1.24^d^	1.81^c^	2.67^b^	2.90^b^	3.28^ab^	3.69^a^
Malic acid	3.15^c^	3.64^b^	4.16^a^	0.00^d^	0.00^d^	0.00^d^
Propionic acid	0.21^d^	0.27^c^	0.40^a^	0.33^b^	0.38^a^	0.40^a^
**Total organic acids**	**5.5**	**7.1**	**8.7**	**17.1**	**18.0**	**17.9**
***Solubilized DM***	***24.7***	***30.4***	***38.4***	***32.5***	***38.5***	***41.2***

HTT temperatures shown in °C. Numbers are presented as weight percentages of solubilized DM, ‘***Solubilized DM’*** is the percentage of DM solubilized during HTT pretreatment, based on mass balance after HTT pretreatment. Different superscript letters ^a,b,c,d^ indicate significantly different values (*P* <0.05) row-wise. DM, dry matter; HTT, hydrothermal treatment.

**Figure 1:**
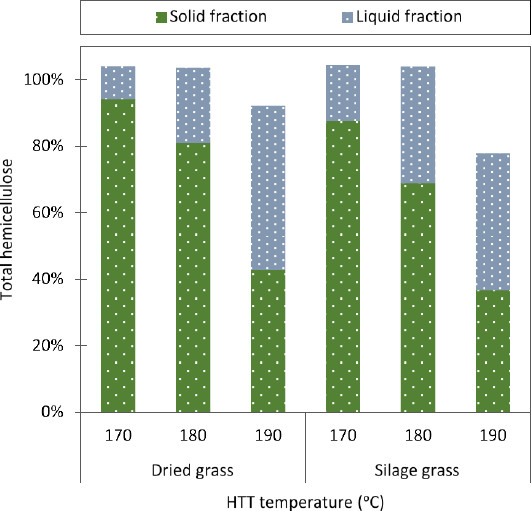
**Recovery of hemicellulose.** Recovered hemicellulose (xylan, arabinan, galactan) (as a percent of total hemicellulose in raw biomass) in solid and liquid fractions after hydrothermal treatment (HTT). Green (dark) dotted columns indicate the recovered levels in the solid fiber fractions, whereas the light blue (light) dotted column sections indicate the hemicellulose levels recovered in the liquid fractions.

The concentration of glucan in the solid fraction and the DM in enzyme hydrolysis are important factors in cellulosic ethanol production as the high viscosity of the fibrous DM fraction determines an upper limit of potential sugars and hence ethanol concentration in the fermentation broth. At bioethanol demonstration plants enzymatic hydrolysis is typically operated at a maximum 30% DM [9]. The higher the glucan concentration in the fiber fraction, the higher the potential ethanol concentration. The relationship between glucan concentration in the solid fraction and potential ethanol concentration mainly applies to the fermentation of C6 sugars only (when the liquid fraction is separated) and the C5 sugars are used for other purposes such as molasses or biogas production [8]. In principle, high solubilization of the biomass is of course an advantage to the enzymatic liquefaction process. The presence of less water insoluble fiber reduces the viscosity of the process stream from the HTT pretreatment and reduces complications of enzymatic hydrolysis at high DM concentrations [26]. However, more severe HTT pretreatments leading to increased biomass solubilization also cause a rise in the concentration of inhibitory compounds.Solubilization of hemicellulose (xylan, arabinan, and galactan) increased as expected at higher HTT temperatures, but there were no differences between dried grass and grass silage in the relative amounts of hemicelluloses in the solid fraction after HTT. Solubilization of hemicellulose was also evident from the calculated hemicellulose recovery from the liquid and solid fractions after HTT (Figure 1). However, the results of hemicellulose recovery (mainly xylose) showed that the actual amount of hemicellulose left in the solid fraction compared to hemicellulose in the raw material differed between the dry grass and grass silage for all HTT temperatures, with the hemicellulose recovery in the solid fraction being lower for the grass silage than for the dry grass after HTT (Figure 1), thus solubilization was higher. The solubilized hemicellulose could be completely recovered from the liquid fraction for HTT at 170 and 180°C. However, at the high temperature HTT of 190°C where the recovery in solid fraction was significantly lower for both biomasses, it was not possible to recover all the hemicellulose (Figure 1). Ensiling and HTT pretreatments therefore brought about further degradation of hemicellulosic monosaccharides due to high severity induced by high temperature, and it was evident that HTT of grass silage resulted in significantly more hemicellulosic monosaccharide degradation than HTT of dried grass.

The high degradation of hemicellulose during HTT of grass silage at 190°C infers that for ensiling to positively contribute to the overall conversion efficacy, the ensiling must give rise to a considerable increase in the enzymatic conversion of the solid fraction to compensate for the loss of WSC and the higher hemicellulose degradation. The improved solubilization and increased degradation of grass silage compared to dry grass after HTT can be explained as follows: firstly, ensiling caused the grass silage to become less recalcitrant than the dry grass, and secondly, the severity of HTT on grass silage was increased by high concentrations of organic acids present in the grass silage. Since changes in the chemical structure of the grasses after ensiling were limited, it is most likely that differences in solubilization and degradation between dry grass and grass silage were primarily due to higher severity of the HTT caused by the presence of high concentrations of organic acids in the grass silage.

Between 12 and 36% of the solubilized DM was recovered as mono- and oligosaccharides and between 6 and 18% were recovered as organic acids (Table 4). The rest of the solubilized DM was presumably made up of proteins, amino acids, and cuticular wax, which were not quantified. Despite the slightly higher solubilization of hemicellulose after HTT of grass silage, it was the HTT pretreated dry grass samples which contained the highest sugar concentrations in the liquid fractions and notably higher amounts of glucose and fructose derived from the WSC (Table 4). The elevated sugar levels in the HTT pretreated dry grass considerably exceeded the surplus of xylose in the liquid fractions of pretreated grass silage (after HTT of 170 and 180°C, respectively) (Table 4). The total amount of organic acids in the liquid fraction were, as expected, significantly higher in the grass silage compared to the dry grass, particularly as a result of the high lactic acid concentration (Table 4) originating from the silage fermentation. The presence of organic acids in the liquid fraction can potentially inhibit ethanol fermentation due to the diffusion of undissociated acids across the yeast cell membrane. Graves *et al*. tested the inhibition caused by both lactic and acetic acids on ethanol fermentation and found inhibitory concentrations at pH 5 and 25% solids, starting from 4.0 (w/v)% and 0.3 (w/v)% of lactic and acetic acids, respectively [27]. Thus lactic acid, which was present at the highest concentrations, is a much less inhibitory acid than acetic acid [27]. The amount of lactic and acetic acids in the liquid fractions contributed around 11-12% and 2.9 to 3.7% of the solubilized DM respectively, corresponding to maximum concentrations of 0.50 and 0.13 (w/v)% respectively in the hydrolysate. Therefore, the organic acid concentrations in the liquid fraction after HTT do not pose a high risk of inhibition of the enzyme hydrolysis or ethanol fermentation.

### Enzymatic hydrolysis

**Figure 2:**
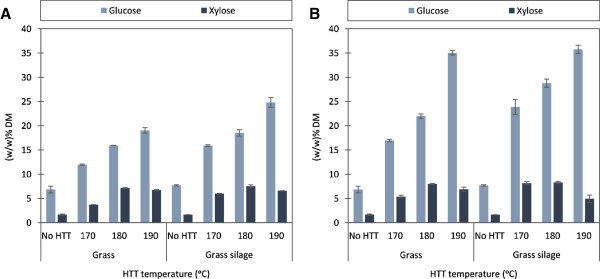
**Glucose and xylose yields after enzymatic hydrolysis of the HTT solid fractions.** Yields are in weight percentages of DM in the HTT solid fraction. **(A)** enzymatic hydrolysis conducted with dried solid fractions and **(B)** enzymatic hydrolysis conducted with washed, wet solid fractions.

Surprisingly, ensiling of grass alone did not improve glucose or xylose yields in the enzymatic hydrolysis. These results contrasted the results obtained in our previous study on grass [18]. Direct enzymatic hydrolysis of grass and grass silage with a mixture of cellulosic and hemicellulosic enzymes (9:1 CTec2: HTec2) produced glucose yields of only approximately 7 (w/w)% (Figure 2A). In our previous study of the same grass species, ensiling directly improved the glucose yield from 7.8 to 11.4 (w/w)%. In the previous study it was concluded that the pretreatment effect was significantly influenced by the DM and biomass composition, which was distinguished by the relative maturity of the grass [18]. The data obtained in the present study supports that the particular grass cut has a significant influence on the effect of ensiling.The enzymatic hydrolysis experiments were performed repeatedly with different sample preparations. When fibers were only dried prior to enzymatic hydrolysis, the amount of released glucose was strikingly low, ranging from 13 to 25 (w/w)% glucose per DM pretreated fiber (Figure 2A), equaling between 29 and 46% cellulose convertibility (converted glucan/original glucan in raw grass). Nevertheless, the results showed increasing glucose yields with increasing HTT temperature as expected, and furthermore, consistently higher glucose yields from the grass silage (Figure 2A) compared to dry grass. Thus, ensiling provided a positive effect on enzymatic cellulose saccharification of the HTT solid fraction.Glucose yields from the enzymatic hydrolysis experiments were unexpectedly low (Figure 2A). The low glucose yields were hypothesized to be due to inhibition of the enzymes and increased recalcitrance induced by the drying of samples following HTT. The enzymatic hydrolysis treatments were therefore repeated twice. Firstly, the samples were washed prior to enzymatic hydrolysis; secondly, samples were washed and subsequently dried prior to enzymatic hydrolysis. Washing without subsequent drying of the HTT fibers gave a significant increase in yields, ranging from 18 to 35 (w/w)% glucose per DM pretreated fiber (Figure 2B). Cellulose convertibility thus increased to between 45 and 78% (converted glucan/original glucan). The glucose yields improved in general by a factor of between 1.4 and 1.5, but for HTT pretreated dry grass at 190°C the improvement was significantly higher, resulting in improved glucose concentrations by a factor of 1.8. Washing and subsequent drying gave similar results as those obtained from washed wet fibers (results not shown). Thus, washing proved to be a necessary step before enzymatic hydrolysis in order to obtain acceptable glucose yields, but the effect of drying was insignificant.

Strong inhibition of cellulases has recently been shown to occur in hydrothermally pretreated biomass, due to the presence of xylo- and gluco-oligosaccharides [28]. Poor glucose yields resulting from the enzymatic hydrolysis of unwashed fibers imply that oligomers from the liquid fraction ‘stick’ to the fibers even after drying, and inhibit cellulases in the enzymatic hydrolysis. Such a high inhibition of cellulases, however, was not found to be an issue for the enzymatic hydrolysis of pretreated wheat straw in our own previous study [22]. Degradation products of furans and acids (furfural, 5-HMF, levulinic acid, and formic acid) derived from degradation of the carbohydrates can also cause cellulase inhibition. These degradation products can form insoluble lignin-like structures that deposit on the pretreated fibers and therefore decrease accessibility for the cellulases [29].

An analysis of the washing waters showed high concentrations of oligosaccharides, which are likely to have caused inhibition of the cellulases in the enzymatic hydrolysis of the unwashed fiber. Kont *et al.*[28] identified inhibitory oligosaccharides consisting of a mixture of xylo- and gluco-oligosaccharides with a degree of polymerization, DP, of 7 to 16, and found them to cause a 100-fold stronger inhibition on biohydrolase *Tr*Cel7A than cellobiose, which is known to be a common inhibitor for cellulases [28].

**Figure 3:**
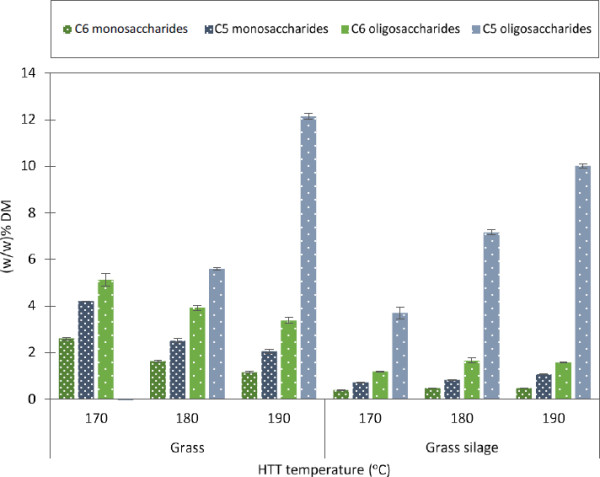
**Mono- and oligosaccharides in the wash water of HTT solid fractions divided in C5 and C6 sugars.** C6 includes (glucose, galactose, and fructose) in (w/w)% of DM pretreated solid fraction. C5 includes (xylose and arabinose) in (w/w)% of DM pretreated solid fraction. DM, dry matter; HTT, hydrothermal treatment.

Washing of the HTT fibers removed considerable amounts of oligosaccharides in all samples and increasing amounts at higher HTT temperatures (Table 5). Sugar analysis of the washing waters showed significant differences between HTT pretreated dry grass and HTT pretreated grass silage (Figure 3). The wash water of HTT pretreated dry grass contained higher amounts of C6 sugars, which decreased with increasing HTT temperature. In contrast, for HTT pretreated grass silage, the concentrations of C6 sugars were lower and constant at all HTT temperatures. The concentrations of oligomer C5 sugars, on the other hand, increased with increasing HTT temperatures for both biomasses (Figure 3).

**Table 5:** Total mono- and oligosaccharides organic acids and furans in wash water of HTT solid fraction

**Biomass**	**Grass**			**Grass silage**		
**HTT temperature**	**170**	**180**	**190**	**170**	**180**	**190**
	**(w/w)% of DM**			**(w/w)% of DM**		
Monosaccharides	6.81^a^ ± 0.07	4.13^b^ ± 0.20	3.19^c^ ± 0.19	1.10^d^ ± 0.07	1.28^d^ ± 0.04	1.53^d^ ± 0.01
Oligosaccharides	5.01^d^ ± 0.43	11.47^c^ ± 0.28	18.20^a^ ± 0.45	5.73^d^ ± 0.45	10.25^c^ ± 0.41	13.46^b^ ± 0.21
C5:C6 ratio of oligomers	na.	1.4	3.6	3.1	4.3	6.4
Organic acids	2.65^b^ ± 0.06	2.94^b^ ± 0.14	2.74^b^ ± 0.22	5.70^a^ ± 0.14	5.81^a^ ± 0.12	5.77^a^ ± 0.03
Furans	0.11^c^ ± 0.00	0.24^b^ ± 0.01	0.46^a^ ± 0.03	0.07^c^ ± 0.00	0.12^c^ ± 0.00	0.21^b^ ± 0.00

HTT temperature presented as °C. Numbers are presented as weight percentages of DM in the HTT solid fraction, followed by standard deviations (^a,b,c,d^). Different superscript letters ^a,b,c,d^ indicate significantly different values (*P* <0.05) row-wise). DM, dry matter; HTT, hydrothermal treatment.

The content of furans in the wash water was higher for the HTT pretreated dry grass than that of grass silage and the major component was 5-HMF. High 5-HMF concentrations in the washing waters of HTT pretreated dry grass can be explained by the production of 5-HMF from degradation of glucose present as free glucose in the WSC of dry grass. The presence of furans in the washing waters suggests that formation and deposits of insoluble pseudo-lignin could also have contributed to cellulase inhibition [29]. Free furans are also potentially inhibitory to the ethanol fermentation [30], but the amounts of free furans in the wash water did not exceed critical levels for inhibition. A solid loading in a subsequent fermentation of 25% w/v would lead to a maximum furan concentration of 0.12 (w/v)%.

A significantly higher concentration of inhibitory oligosaccharides and furans were found in the washing water of the 190°C HTT pretreated dry grass, corroborating the significant effect of washing on cellulase activity for this sample.Washing the HTT pretreated grass silage improved glucose yields at the lower HTT temperatures of 170°C, from 17.0 to 23.8 (w/w)% (45 to 57% cellulose convertibility) and at 180°C from 22.0 to 28.8 (w/w)% (54 to 69% cellulose convertibility) (Figure 2B). HTT pretreatment of grass silage at 170°C gave the same sugar yields as HTT pretreatment of dry grass at 180°C. However, ensiling followed by HTT pretreatment at 190°C had no effect on glucose yields when compared with HTT pretreatment of dry grass at 190°C. At 190°C, HTT pretreatment of dry grass gave the same high glucose yield as the grass silage of 35 (w/w)% (77% cellulose convertibility), and a higher xylan yield of 7 (w/w)% xylose (27% xylan convertibility). It is evident that the lower xylan convertibility for 190°C HTT pretreated grass silage is a direct consequence of increased hemicellulose degradation (Figure 1).

The effect of ensiling of grass prior to HTT is nevertheless consistently smaller than the effect of ensiling prior to HTT observed on wheat straw [22]. Previously, on wheat straw, we thus found that the glucose convertibility of wheat straw was increased by a factor of 1.9 and 1.8 by HTT pretreatment at 170 and 180°C, respectively [22]. The comparable improvement factor for grass was, in both cases, merely 1.3.

Another noticeable difference to the results on wheat straw from [22] was a lower xylose conversion, which for grass is nearly half of that of wheat straw. An explanation for the low xylose conversion observed for grass is the differences between grass and wheat straw hemicellulose and its cross-linkages to lignin, which could have a strong influence on enzymatic hydrolysis. Even though both biomasses are grasses (*Poaceae*) and have hemicellulosic structures that include glucuronoarabinoxylan, xyloglucan, and mixed-linkage glucan, there are also large differences in terms of relative amounts, degree of branching, and cross-linkages [31].

Temperate grasses are, for example, known to have a high degree of ferulate-arabinoxylan cross-links, which have been shown to be a limiting factor for plant cell wall digestion in ruminants [32,33].

It was not within the scope of this study to make detailed structure analysis of the grass, however, such a study could help to identify the reason for the significant differences between the combined ensiling and HTT of grass and wheat straw. Detailed characterization of temperate grass hemicelluloses was conducted by Xu *et al.*[33].

### Total sugars available after pretreatment

**Figure 4:**
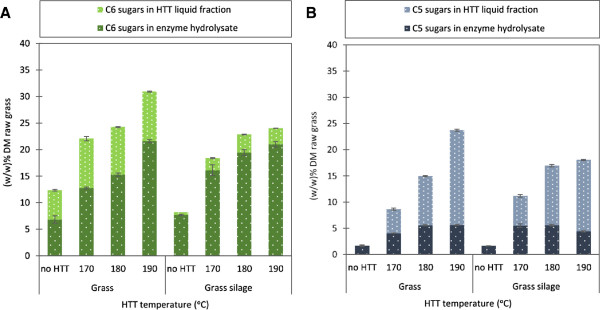
**Released sugars after pretreatment and enzymatic hydrolysis of grass and grass silage. (A)** Total released C6, mono- and oligomers (glucose, galactose, and fructose) as a weight percentage of DM raw grass **(B)** Total released C5, mono- and oligomers (xylose and arabinose) as a weight percentage of DM raw grass in (w/w)% of DM raw grass. Liquid fraction includes sugars from liquid fractions and sugars in wash water of solid fractions. DM, dry matter; HTT, hydrothermal treatment.

To summarize on the overall effect of ensiling prior to HTT, all released sugars derived from the solid and liquid fraction were added together (Figure 4). The sugars in the liquid fraction samples included mono- and oligosaccharides from both the HTT liquid and the wash water.The quantities of C6 sugars clearly showed that ensiling of grass prior to HTT did not give rise to higher amounts of total C6 sugars (Figure 4A); hence, the improved effect of ensiling on enzymatic conversion of cellulose for HTT at 170 and 180°C did not make up for the loss of glucose and fructose associated with ensiling.The total release of C5 sugars were, however, higher for grass silage both for HTT at 170 and 180°C (Figure 4B). This was due to the increased solubilization of hemicellulose but, in the case of HTT 170°C grass silage, also a result of slightly better enzymatic conversion of xylose from the solid fraction. The release of C5 sugars was, however, highest for dry grass pretreated at 190°C.

Overall, the total sugar release results show that at the lower temperatures of 170 and 180°C the total amount of released sugars were exactly the same for dry grass and grass silage (Table 6). In other words, the C6 sugars lost during ensiling was gained as C5 sugars (Figure 4A and 4B). If grass silage is to be used for bioethanol fermentation, fermentation of both C6 and C5 should be included. However, as it was shown in this study, efficient enzymatic hydrolysis required washing of the solid fraction to avoid heavy inhibition of the cellulases. The result of washing prior to enzymatic hydrolysis implies that there could be inhibitors that have to be removed by washing also to ease further conversion beyond enzymatic cellulose hydrolysis. C5 fermenting yeasts are for instance known to have lower inhibitor tolerances than the traditional, commercial *Saccharomyces cerevisiae*[30].

**Table 6:** Total released sugars after pretreatment and enzymatic hydrolysis

**Biomass**	**Grass**			**Grass silage**		
	**(w/w)% of DM**			**(w/w)% of DM**		
**HTT temperature**	**170**	**180**	**190**	**170**	**180**	**190**
Total released sugars	29.5^c^ ± 0.78		39.2^b^ ± 0.61		53.3^a^ ± 0.62	29.6^c^ ± 1.75	39.8^b^ ± 1.03	40.5^b^ ± 0.72

HTT temperature presented as °C. Numbers are presented as weight percentages of DM in the raw grass, followed by standard deviations (^a,b,c,d^). Different superscript values ^a,b,c,d^ indicate significantly different values (*P* <0.05) row-wise. DM, dry matter; HTT, hydrothermal treatment.

Nevertheless, by far the highest overall released sugars were found for HTT pretreated grass at 190°C. Here, ensiling did not improve the enzymatic hydrolysis and furthermore, also caused significant degradation of hemicellulose during HTT.

Further studies including simultaneous saccharification and fermentation of the pretreated biomasses should be conducted in order to investigate the actual effect of inhibition from the liquid fraction and oligomers on the pretreated fibers. Furthermore, the poor enzymatic hemicellulose conversion observed in this study calls for a detailed study of the hemicellulose structure in pretreated grass and identification of structures which could be responsible for the inefficient enzymatic conversion.

## Conclusions

Ensiling of grass prior to HTT resulted in a higher severity of HTT pretreatments which caused increased solubilization and a higher concentration of cellulose in the solid fraction compared to HTT of dry grass. Secondly, ensiling of grass gave rise to an improvement in enzymatic saccharification of both cellulose and hemicellulose at lower HTT temperatures of 170 and 180°C. The improvement was, however, significantly lower than previously found for ensiling of wheat straw. The differences between the effect of ensiling on grass compared to wheat straw is believed to be due to poor hydrolysis of the grass hemicellulose and emphasizes the huge impact of structural differences on biomass processing.

HTT of dry grass and grass silage gave rise to profound inhibition in the enzymatic hydrolysis and washing of pretreated fibers was found to be necessary. Loss of WSC during ensiling is, however, a large drawback for ensiling as effective pretreatment method, and the improvement in pretreatment effect due to ensiling was merely equal or less than the loss of WSC at all HTT temperatures. The overall sugar yield was best for dry grass HTT pretreated at 190°C.

The results in this study prove that ensiling of grass comes with a cost of WSC. The loss of WSC caused by ensiling is not necessarily compensated for by providing a lower temperature requirement for HTT for high enzymatic monosaccharide release. Ensiling does, on the other hand, pose considerable advantages as a storage method.

## Materials and methods

### Raw material

The grass used in the study was *Festulolium* Hykor, which is a crossbreed of the temperate grasses tall fescue (*Festuca arundinacea*) and Italian ryegrass (*Lolium multiflorum*) developed by DLF TRIFOLIUM, Denmark, for high yield potential (18 t ha^−1^) and high persistency throughout the season. *Festulolium* Hykor was harvested on 31.05.2012 (first cut) from a DLF TRIFOLIUM demo plot, sized 1.5 × 8 m and located in southern Zealand, Denmark (55° 20’N, 12° 23’E), with a HALDRUP F-55 harvester (Inotec Engineering GmbH, Ilshofen, Germany). The grass was collected immediately after harvesting and stored in a freezer until use. When thawed, the grass was cut manually to between 10 and 15 cm sized pieces. The chopped grass had a DM content of 26% by weight. The grass was split into two portions, one was air-dried at room temperature and the other was ensiled.

### Pretreatment process

Enzymatic hydrolysis was performed on hydrothermally pretreated ensiled grass and compared to dried grass, grass silage, and hydrothermally pretreated grass.

### Ensiling

The commercially available ensiling inoculum LACTISIL Grass Plus (Chr. Hansen, Hørsholm, Denmark) that consists of freeze-dried pure homofermentative *Pediococcus pentosaceus* and *Lactobacillus plantarum* was applied. A suspension of 0.2 g L^−1^ water was prepared and added (40 mL kg^−1^ grass dry matter) to reach an initial inoculum size of 8 mg kg^−1^; double the amount used previously [20] where the amount was concluded to be ineffectual. Chopped grass weighing 5.8 kg was packed in two layers of thin black polyethyleneterephthalate plastic bags and one layer of thick transparent PET plastic. Anaerobic conditions were achieved by removing the air from the plastic bag using an industrial vacuum cleaner. The plastic bags were opened after four weeks. Weight loss was measured to be 0.6% and was used for the calculation of DM mass balances.

### Hydrothermal pretreatment

Hydrothermal pretreatments (HTT) were carried out in the custom-made ‘Mini IBUS’ equipment (Technical University of Denmark, Risø campus): a pilot plant batch reactor for hydrothermal pretreatment of lignocellulosic biomass (Technical University of Denmark, Lyngby, Denmark) [22]). 1 kg DM (corrected for volatile fatty acid content) of the dried grass and grass silage was treated at various temperatures (170, 180, and 190°C) for 10 minutes. After HTT, the pretreatment reactor was cooled to below 70°C and the material was separated by pressing. Weight and DM was measured for each solid and liquid fraction and used for mass balance calculations. Solid and liquid fractions were kept frozen until further analysis.

### Sample preparation for enzymatic hydrolysis

Enzymatic hydrolysis was done with three different sample preparations on HTT pretreated fibers: (1) using dried and milled (2 mm) fiber, (2) using washed, dried, and milled (2 mm) fiber, and (3) using washed, wet, and cut (10 to 5 mm) fiber. This was done to examine the effect of removing potential inhibitors from the fiber by washing after HTT drying, and to test the influence of drying, which is typically performed during sample preparation in laboratory tests.

### Enzymatic hydrolysis

The enzymatic hydrolysis was performed at 2.0% DM (w/v) in a total volume of 20 ml using a 50 mM citrate buffer at pH 5.0 with 0.4% w/w sodium azide. Commercially available cellulolytic and hemicellulolytic enzyme preparations, Cellic™CTec2 and Cellic™HTec2, from Novozymes A/S (Bagsværd, Denmark) were used in a 9/1 ratio and added to obtain a mixture of 10% enzyme/substrate (w/w cellulose). Cellic™CTec2 is a commercial cellulase preparation based on the cellulase complex produced by *Trichoderma reesei* containing at least two main cellobiohydrolases EC 3.2.1.91 (Cel6A and Cel7A), five different endo-1,4β-glucanases EC 3.2.1.4 (Cel7B, Cel5A, Cel12A, Cel61A, and Cel45A), β-glucosidase EC 3.2.1.21, β-xylosidase EC 3.2.1.37, and GH61 [34], in addition to particular proprietary hydrolysis-boosting proteins. Cellic™HTec2 mainly demonstrates endo-1,4β-xylanase activity EC 3.2.1.8, but also cellulase activity. The enzymatic hydrolysis was performed during shaking for 72 hours at 50°C. Duplicates and enzyme blanks were included. Hydrolysates were analyzed for glucose and xylose levels by high pressure liquid chromatography (HPLC) and the yield is presented per DM fiber in the hydrolysis. Cellulose and xylan convertibility were calculated as the converted cellulose or xylan divided by the original cellulose or xylan content in the grass raw material.

### Chemical analysis

Grass, grass silage, hydrothermally pretreated grass, and hydrothermally pretreated grass silage were analyzed for chemical composition by methods based on standard laboratory analytical procedures developed by National Renewable Energy Laboratory (NREL), United States [35,36]. Deviations from these standard procedures are stated in the following sections. The analysis of the solid fiber fraction included ash content determination, water extraction (only on grass and grass silage), ethanol extraction, and strong acid hydrolysis for structural carbohydrates and lignin. The liquid fractions after HTT and the washing waters from the washing of solid fractions prior to enzymatic hydrolysis, were analyzed directly by HPLC after weak acid hydrolysis to determine oligosaccharide concentration.

### DM determination

DM and ash analyses were performed according to NREL standard laboratory analytical procedures based on oven DM measurements [36]. Since silage biomass contains large amounts of volatile compounds, it is critical to correct the measured oven-DM (at 105°C) for loss of volatiles, to obtain the true DM. The measurements were therefore corrected using concentrations of volatiles and coefficients according to the method by Huida *et al.*[37].

#### Analytical method

Concentrations of carbohydrates (d-glucose, d-xylose, l-arabinose, l-rhamnose, D-galactose, d-mannose and D-fructose) were quantified by HPLC (Shimadzu Corp., Kyoto, Japan) using a HPX-87P column (BioRad, Hercules, California, United States) and refractive index (RI) detection, at 80°C with water as eluent (0.5 ml min^−1^). Organic acids (lactic-, formic-, acetic-, propionic, and butyric acid) were quantified by HPLC using a Biorad HPX-87H column (Hercules, California, United States), RI detection, 63°C and 4 mM H_2_SO_4_ as eluent (0.6 ml min^−1^).

### Water extraction

DM biomass weighing between 0.3 and 0.4 g was extracted from freshly disrupted silage bags in 10 ml Milli-Q H_2_O (Millipore Merck, Darmstadt, Germany) with 10 μl of the antibiotic ampicillin (10 mgml^−1^ solution) to prevent microbial activity during extraction. The extraction samples were shaken for 2 hours at 25°C and 150 rpm. Extracts were analyzed for sugars and acids by HPLC as described above. The amount of water extractives was defined as the mass of material lost through extraction.

### Weak acid hydrolysis

The liquid fraction of HTT and the wash water from the washing of solid fractions prior to enzymatic hydrolysis was analyzed by weak acid hydrolysis to quantify the content of soluble oligomer carbohydrates. HTT liquid fraction measuring 10 ml was autoclaved for 10 minutes at 121°C with 4 w/w% H_2_SO_4_. Derived sugars were analyzed by HPLC as described above.

### Ethanol extraction

Lipophilic extraction was carried out by Soxhlet extraction in a reflux condenser for 6 hours with 99 w/w% ethanol on water extracted samples of grass and grass silage. The amount of ethanol extractives, including volatiles, was defined as the mass of material lost through extraction.

### Determination of structural carbohydrates and lignin

Strong acid hydrolysis was used to measure the carbohydrate and lignin content of the extracted bio residue based on the NREL standard laboratory analytical procedure [35].

### Statistics

One-way analyses of variances of all analytical data (one-way ANOVA): 95% confidence intervals were compared as Tukey-Kramer intervals calculated from pooled standard deviations (Minitab Statistical Software, Addison-Wesley, Reading, Massachusetts, United States).

## Abbreviations

DM: Dry matter; HPLC: High-performance liquid chromatography; HTT: Hydrothermal treatment; LAB: Lactic acid bacteria; WSC: Water soluble carbohydrates.

## Competing interests

The authors declare that they have no competing interests.

## Authors’ contributions

All authors participated in the design and data interpretation of the study. MA-J conducted the experiments and drafted the manuscript. KSJ and TD contributed with materials and helped design the study. MA-J, ZK, and AM designed the research and analyzed and interpreted the data. MA-J and AM wrote the paper. All authors read and approved the final manuscript.

## References

[B1] DIRECTIVE 2009/28/EC OF THE EUROPEAN PARLIAMENT AND OF THE COUNCIL of 23 April 2009 on the promotion of the use of energy from renewable sources and amending and subsequently repealing Directives 2001/77/EC and 2003/30/EC [2009] OJ L 140 5.6.2009 16

[B2] Proposal for a DIRECTIVE OF THE EUROPEAN PARLIAMENT AND OF THE COUNCIL amending Directive 98/70/EC relating to the quality of petrol and diesel fuels and amending Directive 2009/28/EC on the promotion of the use of energy from renewable sources 2012 Brussels 17.10.2012 COM(2012) 595 final 2012/0288 (COD)

[B3] Allen BR Keegan D Elbersen B Biomass and bioenergy in the wider land-use context of the European Union Biofuels Bioprod Bioref 2013 7 2 207 216

[B4] Tilman D Hill J Lehman C Carbon-negative biofuels from low-input high-diversity grassland biomass Science 2006 314 5805 1598 1600 17158327 10.1126/science.1133306

[B5] Buxton DR Muck RE Harrison JH Silage science and technology 2003 Wisonsin: American Society of Agronomy, Inc. Crop Science Society of America, Inc. Soil Science Society of America, Inc

[B6] Rooke JA Hatfield RD Buxton DR, Muck RE, Harrison JH Biochemistry of ensiling Silage science and technology 2003 Wisconsin: American Society of Agronomy, Crop Science Society of America, Soil Science Society of America 95 140

[B7] Yang HY Wang XF Liu JB Gao LJ Ishii M Igarashi Y Cui ZJ Effects of water-soluble carbohydrate content on silage fermentation of wheat straw J Biosci Bioeng 2006 101 3 232 237 16716924 10.1263/jbb.101.232

[B8] Ren H Richard TL Moore KJ The impact of enzyme characteristics on corn stover fiber degradation and acid production during ensiled storage Appl Biochem Biotechnol 2007 137–140 1–12 221 238 10.1007/s12010-007-9054-2 18478391

[B9] Larsen J Haven MT Thirup L Inbicon makes lignocellulosic ethanol a commercial reality Biomass Bioenergy 2012 46 36 45

[B10] Zhang PF Zhang Q Pei ZJ Wang DH Cost estimates of cellulosic ethanol production: a review J Manuf Sci Eng 2013 135 2 021005

[B11] Festel G Würmseher M Rammer C Boles E Bellof M Modelling production cost scenarios for biofuels and fossil fuels in Europe J Clean Prod 2014 66 242 253

[B12] Yang B Wyman CE Pretreatment: the key to unlocking low-cost cellulosic ethanol Biofuels Bioprod Bioref 2008 2 1 26 40

[B13] Bals B Wedding C Balan V Sendich E Dale B Evaluating the impact of ammonia fiber expansion (AFEX) pretreatment conditions on the cost of ethanol production Bioresour Technol 2011 102 2 1277 1283 20826086 10.1016/j.biortech.2010.08.058

[B14] Chen Y Sharma-Shivappa RR Chen C Ensiling agricultural residues for bioethanol production Appl Biochem Biotechnol 2007 143 1 80 92 18025598 10.1007/s12010-007-0030-7

[B15] Digman MF Shinners KJ Casler MD Dien BS Hatfield RD Jung H-JG Muck RE Weimer PJ Optimizing on-farm pretreatment of perennial grasses for fuel ethanol production Bioresour Technol 2010 101 14 5305 5314 20202834 10.1016/j.biortech.2010.02.014

[B16] Oleskowicz-Popiel P Thomsen AB Schmidt JE Ensiling - wet-storage method for lignocellulosic biomass for bioethanol production Biomass Bioenergy 2011 35 5 2087 2092

[B17] Pakarinen A Maijala P Jaakkola S Stoddard F Kymäläinen M Viikari L Evaluation of preservation methods for improving biogas production and enzymatic conversion yields of annual crops Biotechnol Biofuels 2011 4 1 20 21771298 10.1186/1754-6834-4-20 PMC3155480

[B18] Ambye-Jensen M Johansen KS Didion T Kádár Z Schmidt JE Meyer AS Ensiling as biological pretreatment of grass (*festulolium* hykor): the effect of composition, dry matter, and inocula on cellulose convertibility Biomass Bioenergy 2013 58 303 312

[B19] Galbe M Zacchi G Pretreatment: the key to efficient utilization of lignocellulosic materials Biomass Bioenergy 2012 46 70 78

[B20] Pedersen M Meyer AS Lignocellulose pretreatment – understanding biomatrix opening in relation to temperature and pH during pretreatment New Biotechnol 2010 27 6 739 750 10.1016/j.nbt.2010.05.003 20460178

[B21] Hansen MAT Kristensen JB Felby C Jørgensen H Pretreatment and enzymatic hydrolysis of wheat straw (*Triticum aestivum* L.) - the impact of lignin relocation and plant tissues on enzymatic accessibility Bioresour Technol 2011 102 3 2804 2811 21036603 10.1016/j.biortech.2010.10.030

[B22] Ambye-Jensen M Thomsen ST Kádár Z Meyer AS Ensiling of wheat straw decreases the required temperature in hydrothermal pretreatment Biotechnol Biofuels 2013 6 1 116 23945109 10.1186/1754-6834-6-116 PMC3751596

[B23] Dewar WA McDonald P Whittenbury R The hydrolysis of grass hemicelluloses during ensilage J Sci Food Agric 1963 14 6 411 417

[B24] Morrison IM Changes in the lignin and hemicellulose concentrations of 10 varieties of temperate grasses with increasing maturity Grass Forage Sci 1980 35 4 287 293

[B25] Pahlow G Muck RE Driehuis F Elferink SJWHO Spoelstra SF Buxton DR, Muck RE, Harrison JH Microbiology of ensiling Silage science and technology 2003 Wisconsin: American Society of Agronomy, Crop Science Society of America, Soil Science Society of America 31 94

[B26] Jørgensen H Vibe-Pedersen J Larsen J Felby C Liquefaction of lignocellulose at high-solids concentrations Biotechnol Bioeng 2007 96 5 862 870 16865734 10.1002/bit.21115

[B27] Graves T Narendranath NV Dawson K Power R Effect of pH and lactic or acetic acid on ethanol productivity by *Saccharomyces cerevisiae* in corn mash J Ind Microbiol Biotechnol 2006 33 6 469 474 16491359 10.1007/s10295-006-0091-6

[B28] Kont R Kurašin M Teugjas H Väljamäe P Strong cellulase inhibitors from the hydrothermal pretreatment of wheat straw Biotechnol Biofuels 2013 6 1 135 24053778 10.1186/1754-6834-6-135 PMC3849272

[B29] Kumar R Hu F Sannigrahi P Jung S Ragauskas AJ Wyman CE Carbohydrate derived-pseudo-lignin can retard cellulose biological conversion Biotechnol Bioeng 2013 110 3 737 753 23042575 10.1002/bit.24744

[B30] Klinke HB Thomsen AB Ahring BK Inhibition of ethanol-producing yeast and bacteria by degradation products produced during pre-treatment of biomass Appl Microbiol Biotechnol 2004 66 1 10 26 15300416 10.1007/s00253-004-1642-2

[B31] Scheller HV Ulvskov P Hemicelluloses Annu Rev Plant Biol 2010 61 263 289 20192742 10.1146/annurev-arplant-042809-112315

[B32] Yu P McKinnon JJ Christensen DA Hydroxycinnamic acids and ferulic acid esterase in relation to biodegradation of complex plant cell walls Can J Anim Sci 2005 85 3 255 267

[B33] Xu F Geng ZC Sun JX Liu CF Ren JL Sun RC Fowler P Baird MS Fractional and structural characterization of hemicelluloses from perennial ryegrass (*Lolium perenne*) and cocksfoot grass (*Dactylis glomerata*) Carbohydr Res 2006 341 12 2073 2082 16750181 10.1016/j.carres.2006.04.033

[B34] Quinlan RJ Sweeney MD Lo Leggio L Otten H Poulsen J-CN Johansen KS Krogh KBRM Jørgensen CI Tovborg M Anthonsen A Tryfona T Walter CP Dupree P Xu F Davies GJ Walton PH Insights into the oxidative degradation of cellulose by a copper metalloenzyme that exploits biomass components Proc Natl Acad Sci USA 2011 108 37 15079 15084 21876164 10.1073/pnas.1105776108 PMC3174640

[B35] Sluiter A Hames B Ruiz R Scarlata C Sluiter J Templeton D Crocker D Determination of structural carbohydrates and lignin in biomass NREL Technical Report 2011 NREL/TP-510-42618 (Version 07.08.2011). National Renewable Energy Laboratory; 1 (Boulder, Colorado)

[B36] Sluiter A Hames B Hyman D Payne C Ruiz R Scarlata C Sluiter J Templeton D Wolfe J Determination of total solids in biomass and total dissolved solids in liquid process samples NREL Technical Report 2008 NREL/TP-510-42621 (Version 03.31.2008). National Renewable Energy Laboratory; 1. (Boulder, Colorado)

[B37] Huida L Väätäinen H Lampila L Comparison of dry-matter contents in grass silages as determined by oven drying and gas-chromatographic water analysis Ann Agr Fen 1986 25 3 215 230

